# Gained social identification and sense of shared experience after flood exposure: Psychosocial pathways to post‐traumatic symptoms and growth

**DOI:** 10.1111/aphw.70193

**Published:** 2026-07-18

**Authors:** Magdalena Skrodzka, Sabina Toruńczyk‐Ruiz, Anna Wnuk

**Affiliations:** ^1^ School of Psychology University of Queensland Brisbane Queensland Australia; ^2^ Faculty of Psychology University of Warsaw Warsaw Poland

**Keywords:** flood, post‐traumatic growth, post‐traumatic symptoms, sense of shared experience, social identity

## Abstract

This research provides the first quasi‐experimental and longitudinal examination of the Social Identity Model of Traumatic Identity Change among individuals affected by the September 2024 flood in Poland. Advancing the state of the art, we analyzed the emergence of identification with flood‐affected people and extended the model by incorporating the sense of shared experience (SSE) within this group. Surveys were completed 3 months *(N* = 630) and 7 months after the flood *(N* = 315).

The results indicated that the severity of flood exposure was linked with higher post‐traumatic stress (PTS) and post‐traumatic growth (PTG). Repeated trauma strengthened both identification and SSE with those affected by the flood. Over time, SSE remained stable, whereas identification decreased, suggesting that SSE serves as a more enduring psychosocial resource than group identification. SSE predicted the subsequent PTG but not the PTS, highlighting its positive role in adaptive coping, while PTS contributed to later increases in SSE. Multi‐group analyses comparing participants from flood‐prone areas who were at risk but had not experienced flooding, those with less recent flood exposure, those with recent flood exposure, and those experiencing re‐traumatization from both past and recent floods revealed that the pathways linking identification, SSE, and post‐traumatic outcomes differed across these groups. These findings demonstrate the differential function of social processes under varying trauma contexts. Overall, this research underscores the importance of considering exposure history in post‐disaster interventions and highlights how social identity and shared experience can both support resilience and shape recovery trajectories.

## INTRODUCTION

Floods represent one of the most common and globally disruptive natural hazards, increasingly linked to climate change, with wide‐ranging health and psychosocial consequences. Just between 1990 and 2022, 4713 flood events were recorded, affecting over 3.2 billion people worldwide (Liu et al., 2024). Their impacts extend far beyond physical damage and mortality (Lynch et al., 2025), as floods have been consistently shown to undermine psychological well‐being (Fontalba‐Navas et al., 2017; Golitaleb et al., 2022). Evidence further indicates that repeated flood exposure amplifies the psychological burden, with survivors of multiple floods reporting higher distress and lower quality of life compared to those affected once (French et al., 2019). Importantly, ill‐health symptoms are not the sole outcome of flood exposure. Survivors also report post‐traumatic growth (PTG)–positive psychological change such as renewed meaning or strengthened social ties (Calhoun & Tedeschi, 2006; Warsini et al., 2022). With climate change intensifying the frequency and severity of extreme weather events (Dharmarathne et al., 2024), understanding the psychosocial consequences of flooding is a pressing public health concern.

Crucially, the significance of floods as traumatic experiences lies not only in their individual impact but also in their social nature, where identification with others plays a pivotal role in shaping psychological trajectories. The Social Identity Model of Traumatic Identity Change (SIMTIC; Muldoon et al., 2019), proposes that traumatic events can disrupt and also transform social identities, with new group memberships providing a basis for meaning, belonging, and psychological recovery. Although much of the empirical evidence supporting the SIMTIC has focused on traumatic events caused by other people, natural disasters—often experienced collectively—can also foster processes of social identification. In line with this perspective, experiencing a flood may promote identification with others who have undergone similar adversity. Testimonies from flood‐affected individuals highlight the importance of perceiving a shared experience when processing traumatic events (Ntontis et al., 2020; Quinn et al., 2020). This perceived sense of shared experience (SSE) may therefore represent an important psychosocial mechanism linking collective adversity to post‐traumatic outcomes. However, there is a notable lack of quantitative research examining the emergence of newly formed social identities and a SSE among populations affected by flooding. Such research could help clarify how collective disasters shape both post‐traumatic symptoms (PTS) and PTG.

To address this gap, the present study investigates the psychosocial pathways to post‐traumatic outcomes among Polish residents with varying degrees of exposure to the major flood events. Poland provides a particularly informative context for examining these processes because the country has experienced several severe floods over the past decades, allowing researchers to examine how both recent and prior disaster exposure may shape post‐traumatic responses. In particular, the catastrophic 1997 flood and the recent 2024 flood offer an opportunity to study the psychological consequences of a large‐scale disaster in a context where collective memory of earlier flooding may still be present.

The first of these floods, which took place in July 1997, was a catastrophic flood often referred to as the “Millennium Flood”. It affected large parts of Central Europe, with Poland— in particular its southern and western region, especially Wrocław—among the hardest‐hit countries. In Poland, 56 people died directly as a result of the flood, and an additional 20 deaths by suicide were reported during this period (Czabański, 2005). More than 140,000 residents lost their homes, and approximately 665,000 ha were inundated, causing widespread destruction of infrastructure, housing, and agricultural land (Grela et al., 1999). Studies conducted among individuals affected by the 1997 flood documented substantial and long‐lasting psychological consequences. For instance, Strelau and Zawadzki (2005) found high levels of post‐traumatic stress disorder (PTSD) symptoms both 3 and 15 months after the disaster, with symptoms remaining elevated even 3 years later. Another study conducted more than 5 years after the event reported that over 30% of flood‐affected individuals met clinical criteria for PTSD (Stępień et al., 2005). Since 1997, Poland has experienced several smaller floods, notably in 2001 and 2010, although their overall extent was considerably less severe.

In September 2024, Poland again faced severe flooding in the southwestern regions. Over several days in mid‐September, rainfall reached approximately 118% of the monthly norm, resulting in estimated damages of around USD 3.63 billion (Państwowe Gospodarstwo Wodne Wody Polskie [PGWWP], 2025). More than 238,000 residents were affected, with nine confirmed fatalities and extensive losses to housing, public institutions, and infrastructure (PGWWP, 2025). Early evidence also suggests acute psychological consequences of these floods: a study of 107 directly affected residents revealed that 70% met the criteria for PTSD, 82% reported perceiving their lives to be at risk, and 15% reported health damage following the flood (Prochnij et al., 2025).

Taken together, the recurrence of major flood events in Poland provides a valuable context for examining how disaster exposure relates to post‐traumatic outcomes and the psychosocial mechanisms that shape these responses. By comparing first‐time and repeat survivors and employing a longitudinal design, we examined how flood experiences shape social identification and SSE, and how these processes, in turn, relate to PTS and growth over time. Integrating social identity theory with longitudinal mental health assessment, this study provides novel, evidence‐based insights into the psychosocial mechanisms that foster recovery and adaptation following collective traumatic events, contributing to both disaster psychology and public health scholarship.

## FLOOD EXPOSURE AND POST‐TRAUMATIC OUTCOMES

Exposure to flood is listed as one of the potential traumatic experiences (see Carlson et al., 2005; Hooper et al., 2011). Indeed, evidence from multiple countries demonstrates that floods can have a profound and lasting impact on the health of those affected. For instance, a meta‐analysis of 23 studies conducted across eight countries between 2015 and 2021 found that 29.5% of participants exhibited PTS that could be classified as PTSD (Golitaleb et al., 2022). In a study by Zavala et al. (2023) conducted 1 year after the flood, 30.4% of the 1077 respondents met the criteria of PTSD. It has been suggested that individuals exposed to severe flood have even an eight‐fold elevated risk of developing PTSD compared to residents of nearby localities not exposed to the flood (Fontalba‐Navas et al., 2017). Additionally, mental ill‐health symptoms may persist for several years after the event, highlighting the long‐term psychological burden of flood exposure (Alderman et al., 2012; Apel & Coenen, 2021).

At the same time, exposure to floods can give rise to PTG, reflecting positive psychological changes that follow traumatic experiences (Calhoun & Tedeschi, 2006). Among Colorado flood survivors, the search for meaning in life but not when combined with the presence of meaning showed consistent relationships with PTG (Dursun et al., 2016). A study from Pakistan by Aslam and Kamal (2015) found that PTG among flood‐affected people was predicted by the use of adaptive coping strategies, including active problem solving, positive reframing, and acceptance. Nevertheless, PTG and PTS are not mutually exclusive and can co‐occur, as supported by evidence from flood‐affected populations (e.g., Aslam & Kamal, 2015; Dursun et al., 2016; Warsini et al., 2022; Zavala et al., 2023).

The exposure to flooding, its severity, and the possibility of re‐experiencing it may also be determinants of post‐traumatic health outcomes. Those who experienced flood compared to those “at‐risk” show more mental ill‐health symptoms (e.g., Bei et al., 2013; French et al., 2019; Tunstall et al., 2006). Research also suggests that individuals who have previously experienced flooding report more severe anxiety and PTS when exposed to subsequent flood events, compared to those experiencing flooding for the first time, indicating the risk of re‐traumatization (Mason et al., 2010). In an Indonesian sample, the strength of PTG did not depend on the mere experience of a flood but on its severity and the strength of post‐flood PTS symptoms (Warsini et al., 2022). A study conducted in the United States comparing participants who were not affected by the flood, those with a single recent exposure, and those who experienced flooding twice revealed that participants who had not been affected by the flood exhibited lower symptoms of PTS and depression than flood‐affected individuals (Cherry et al., 2023). Over time, PTS remained stable among non‐affected participants but significantly decreased in both groups exposed to flooding. Interestingly, a reduction in depression symptoms over time was observed only in participants with a single flood exposure (Cherry et al., 2023). We therefore expected that PTS and PTG would be most prevalent among individuals who experienced severe re‐traumatization.

The relationship between flood exposure and social identification or SSE remains less clear. Some studies suggest that the sense of local community may be independent of the severity of the flood (Chang, 2010). At the same time, repeated exposure to traumatic events may strengthen feelings of shared experience (see, e.g., Drury et al., 2019; Vezzali et al., 2016). Thus, we refrain from formulating directional hypotheses regarding the relationship between flood exposure and both changes in identification and SSE and instead address this issue as a research question.

## SOCIAL IDENTITY AND SSE AS PSYCHOSOCIAL PATHWAYS TO POST‐TRAUMATIC SYMPTOMS AND GROWTH

Qualitative research by Ntontis et al. (2018) demonstrates that shared identification with flood‐affected people extends beyond those directly exposed, encompassing wider community members who empathize with them and engage in the recovery process. This suggests that, beyond the impact of individual‐level exposure, social identity and a SSE may serve as key psychosocial pathways shaping both PTS and PTG in the aftermath of flooding. In the studied context, identification with flood‐affected people refers to a perceived sense of belonging to a group defined by shared adversity, whereas a SSE captures the perception of commonality, mutual understanding, and collective meaning‐making among those who have endured the event.

Testimonies collected by Quinn et al. (2020) after a flood provide evidence of social identities evolving after the event, with survivors reporting the emergence of new collective identities—an observation consistent with SIMTIC (Muldoon et al., 2019). The SIMTIC highlights how traumatic events can disrupt existing identities and also open opportunities for new social identifications. Additionally, Muldoon et al. (2019) propose that gaining new social identities because of trauma may decrease PTS and increase the likelihood of PTG. Empirical evidence supports these processes. For instance, Craig et al. (2024) showed experimentally that gaining new group memberships after a flood relates to higher, for example, appreciation of community (social identity revitalization), which, in turn, predicts greater PTG. However, this study did not examine the specific content of new social identities, instead asking participants to list a range of groups they had joined following the flood. Related research with refugees fleeing from war by Skrodzka et al. (2024) underscores the importance of *which* identities are gained: identification with host societies was associated with lower PTS and greater PTG, whereas a stronger refugee identity predicted higher PTS. Interestingly, when a SSE with other refugees was taken into account, refugee identity was linked to greater PTG but remained unrelated to PTS (Skrodzka et al., 2024). Qualitative findings from flood‐affected communities further illustrate these dynamics. Those affected by flood community described the gaining of new social identities as helping to frame personal experience and reduce the isolation of individual suffering by transforming it into a collectively shared experience (Quinn et al., 2020). A SSE has also been reported to transform social norms, encouraging greater openness and reciprocity among community members (Quinn et al., 2020). Other testimonies suggest that maintaining newly formed identities depends on the durability of bonds created during shared adversity, highlighting the reciprocal relationship between identification and shared experience (Ntontis et al., 2020). Yet, despite the growing body of qualitative and experimental evidence, a significant gap remains in quantitative research investigating how identification and a SSE with flood‐affected groups relate to post‐traumatic outcomes in disaster contexts. Nevertheless, based on theory and previous studies mentioned earlier, we expected that over time, identification, and SSE would mutually reinforce one another. Finally, higher levels of identification and SSE should be associated with lower PTS and higher PTG.

## THE PRESENT STUDY

The present study examines how social identification and a SSE with flood‐affected people relate to PTS and PTG following flood exposure. The study, conducted in Poland, draws on participants who were affected by major floods in 1997 and 2024, including both first‐time and repeat flood survivors. The study leverages this unique context to explore both the short‐ and long‐term consequences of disaster exposure.

Building on the SIMTIC (Muldoon et al., 2019) and qualitative evidence highlighting the role of collective experience in shaping recovery after disasters (Ntontis et al., 2018; Quinn et al., 2020), we consider two psychosocial resources that may emerge in the aftermath of floods: (1) identification with flood‐affected people and (2) a sense of shared experience. Both processes are theorized to influence post‐traumatic outcomes by providing social support, meaning, and opportunities for collective coping.

Based on prior research and theory, we hypothesized that post‐traumatic outcomes would differ depending on flood exposure. We predicted that PTS (H1a) and PTG (H1b) would be most prevalent among individuals who experienced severe re‐traumatization. Over time, identification with and SSE with those affected by a flood would be expected to mutually reinforce one another (H2). Finally, higher levels of identification with those affected by a flood (H3) and SSE with those affected by a flood (H4) would be associated with lower PTS and higher PTG, highlighting their protective and growth‐promoting roles in the aftermath of floods. Additionally, informed by previous research (e.g., Cherry et al., 2023), we explored possible differences in the longitudinal relations of psychosocial pathways to PTS and PTG depending on the flood exposure and examined whether identification and SSE vary depending on the severity of flood exposure.

## METHOD

### Procedure

Participants were recruited through social media and an external research agency (Pollster), targeting residents of 45 counties in Poland where a state of natural disaster was officially declared in September 2024. On social media, advertisements of the study were posted with a link directing participants to the online questionnaire. Recruitment via Pollster, a platform with over 450,000 panel members, involved targeted sampling based on participants' places of residence. All participants received detailed information about the study and provided informed consent before participating.

The first wave of data collection (T1) was conducted between December 2024 and January 2025, which was 3 months after the flood. Of the initial sample, 630 participants (of which 73 were recruited via social media and 555 via the research agency) were invited to take part in the second wave (T2). The participants recruited via social media who agreed to be re‐contacted for follow‐up provided their email addresses. To preserve anonymity, these addresses were stored separately from the survey responses. Only one of the authors had access to link the data using participant codes. The participants recruited through the Pollster platform were contacted directly by the company, and the authors were not involved in this process. The second data collection occurred between April and May 2025, yielding responses from 315 participants. The participants recruited via social media received voucher codes for a major supermarket, whereas Pollster panel participants received reward points through the platform.

### Participants

The first wave of data collection included a total of 630 participants whose ages ranged from 18 to 78 years (*M* = 43.34, *SD* = 14.51). Of these, 402 (63.81%) individuals identified as women, and 228 (36.19%) as men. In our analyses, the participants were categorized into five groups based on their flood exposure history (see *Measures*). The first group consisted of residents who reported no experience of their town being flooded in 2024 or 1997—the “at risk only” group (*N* = 140, 22.22%). The second group included residents whose town experienced the 1997 flood but not the 2024 flood—the “at‐risk with past exposure” group (*N* = 85, 13.49%). The third group comprised residents whose town experienced the 2024 flood but not the 1997 flood—the “recent exposure only” group (*N* = 27, 4.29%). The fourth and fifth groups consisted of residents whose towns experienced flooding in both 1997 and 2024 but differed in the severity of their exposure. Those with a smaller level of impact were classified as experiencing “mild re‐traumatization” (*N* = 197, 31.27%), whereas individuals with a greater level of exposure were categorized as experiencing “severe re‐traumatization” (*n* = 181, 28.73%). A detailed description of the demographic characteristics across the five groups is presented in Table 1.

**TABLE 1:** Demographic characteristics across the five groups at two time points.

	At‐risk only	At‐risk with past exposure	Recent exposure only	Mild re‐traumatization	Severe re‐traumatization
T1					
Total sample *n* (%)					
	140 (22.22)	85 (13.49)	27 (4.29)	197 (31.27)	181 (28.73)
Gender *n* (%)					
Women	84 (60.00)	53 (62.35)	19 (70.37)	120 (60.91)	126 (69.61)
Men	56 (40.00)	32 (37.65)	8 (29.63)	77 (39.09)	55 (30.39)
Age *M* (*SD*)					
	43.21 (14.43)	44.86 (15.52)	43.41 (14.44)	44.03 (14.66)	41.98 (13.96)
T2					
Total sample *n* (%)					
	65 (20.63)	50 (15.87)	17 (5.40)	67 (21.27)	116 (36.83)
Gender *n* (%)					
Women	32 (49.23)	27 (54.00)	11 (64.71)	33 (49.25)	77 (66.38)
Men	33 (50.77)	23 (46.00)	6 (35.29)	34 (50.75)	39 (33.62)
Age *M* (*SD*)					
	46.74 (15.27)	49.30 (15.81)	43.53 (16.30)	48.63 (14.85)	44.42 (14.43)

The follow‐up study (T2) included 315 participants from the first wave. The sample included 180 (57.14%) individuals who were identified as women, and 135 (42.86%) who were identified as men, with an average age of 46.52 (*SD* = 14.51; min. 18, max. 78). The “at risk only” group included 65 (20.65%) participants, the “at‐risk with past exposure” group comprised 50 (15.87%) participants, and the “recent exposure only” group included 17 (5.40%) participants. The “mild re‐traumatization” group consisted of 67 (21.27%) participants, whereas the “severe re‐traumatization” group included 161 (36.83%) participants. Further demographic details for T2 participants are presented in Table 1. Comparisons of study variables between participants who completed the follow‐up study and those who did not are presented in Table S1.

### Measures

#### Identification with flood‐affected people

The participants' identification with flood‐affected people was assessed at both time points using a single‐item measure based on Postmes et al. (2013). The participants responded to the statement: *I identify with flood‐affected people*, on a scale ranging from 1 (*strongly disagree*) to 7 (*strongly agree*). Higher scores indicated a stronger identification with flood‐affected people.

#### SSE with flood‐affected people

The participants responded to four items (e.g., *People affected by the flood share my experiences, emotions, beliefs, struggles*) adapted from Skrodzka et al. (2024) to measure the SSE with flood‐affected people at both time points. Responses were given on a scale from 1 (*strongly disagree*) to 7 (*strongly agree*). SSE with flood‐affected people scores were calculated as the mean of the four items (T1: *α* = 0.96, T2: *α* = 0.96). Higher scores indicated a greater SSE with flood‐affected people.

#### Post‐traumatic symptoms (PTS)

Post‐traumatic symptoms related to flood experience were assessed by using nine items from the International Trauma Questionnaire (Cloitre et al., 2018). At both time points, the participants indicated, on a scale from 0 (*not at all*) to 4 (*extremely*), whether they had experienced specific symptoms in the past month, with six items assessing symptom presence (e.g., *Being “super‐alert,” watchful, or on guard*), and three items assessing the impact of these symptoms on daily life (e.g., *Affected your work or ability to work*). Post‐traumatic symptoms scores were calculated as the mean score of the nine items (T1: *α* = 0.94, T2: *α* = 0.94). Higher scores indicated a greater severity of PTS.

#### Post‐traumatic growth (PTG)

PTG following flood experience was assessed using 10 items adapted from Cann et al. (2010). The participants indicated the extent to which they experienced positive psychological changes as a result of the flood (e.g., *I changed my priorities about what is important in life*) on a scale ranging from 0 (*I did not experience this change*) to 5 (*I experienced this change to a very great degree*). PTG scores were calculated as the mean of the 10 items (T1: *α* = 0.94, T2: *α* = 0.93). Higher scores reflected greater PTG.

#### Flood exposure

The participants were asked whether they experienced the two major floods in Poland with the following questions: (1) *To what extent was your town of residence flooded during the floods in September 2024?* (2) *Was your town of residence flooded in 1997?* Responses to both questions were given on a scale from 0 (*not at all*) to 10 (*completely*). Based on responses to these two questions, we created a variable classifying the participants into five groups: (1) those who reported no experience of flooding in 2024 or 1997 (the “at‐risk only” group); (2) those who experienced the 1997 flood but not the 2024 flood (the “at‐risk with past exposure” group); (3) those who experienced the 2024 flood but not the 1997 flood (the “recent exposure only” group); (4) those who experienced flooding in both 1997 and 2024 at a smaller level of impact, that is, a combined score below 9 (the “mild re‐traumatization” group); and (5) those who experienced flooding in both 1997 and 2024 at a greater level of exposure, that is, with a combined score of at least 9 (the “severe re‐traumatization” group).

#### Demographic characteristics

We also measured participants' gender (0 = *female*, 1 = *male*, 2 *= other*, 3 *= prefer not to say*) and age (in years).

### Overview of analyses

First, probable PTSD was assessed following the scoring guidance of the International Trauma Questionnaire (ITQ; Cloitre et al., 2018). The participants were classified as having probable PTSD if they endorsed at least one symptom rated “moderately” (≥ 2) in each of the three PTSD clusters (re‐experiencing, avoidance, sense of threat) and at least one functional impairment item scored ≥2.

To examine how flood exposure influenced psychosocial and post‐traumatic outcomes, one‐way analyses of variance (ANOVAs) were conducted on mean scores of variables at T1. Post hoc comparisons were performed using the Games‐Howell test to account for unequal variances between groups. Next, a mixed factorial multivariate analysis of variance (MANOVA) with repeated measures was conducted to investigate the effects of flood exposure and time on the combined set of dependent variables, allowing assessment of both main effects and interactions.

Two longitudinal analyses were conducted using cross‐lagged panel models to examine temporal relationships among social identification, SSE, and post‐traumatic outcomes at the two measurement points. The first model employed a multi‐group cross‐lagged path analysis to investigate whether the relationships between our variables of interest varied across groups with varying levels of flood exposure. The second model assessed the relationships between the key variables in the overall sample, with flood exposure as a control variable. This model therefore provides a baseline test of the hypothesized relationships in the broader flood‐affected sample. These detailed results of this model are presented in the see Tables S2 and S3.

The multi‐group model produced group‐specific parameter estimates while providing a single overall model fit. Both path models were estimated in AMOS 29 using bootstrapping (10,000 samples) with 95% confidence interval. Model fit was evaluated using commonly recommended indices, with acceptable thresholds defined as a relative chi‐square (*χ* ^2^/*df*) close to 2 (Tabachnick & Fidell, 2007), a root mean square error of approximation (RMSEA) below 0.08 (MacCallum et al., 1996), a comparative fit index (CFI) above 0.90 (Marsh & Hau, 1996), and a standardized root mean square residual (SRMR) below 0.08 (Hu & Bentler, 1999).

## RESULTS

### Post‐traumatic outcomes differences across flood exposure groups

#### T1

From the total sample of 630 participants, 10 individuals did not complete the PTS items. Among the remaining 620 participants, 217 (35.00%) met the criteria for probable PTSD. The prevalence rate significantly differed across the five flood exposure groups, *F*(4,615) = 8.23, *p* < .001 (see Table 2). The severe re‐traumatization group showed the highest prevalence, with 89 participants (51.74%) meeting the criteria. Post‐hoc comparisons using Games–Howell test showed no significant difference between this group and the mild re‐traumatization group. Mean levels of PTS and PTG also differed across groups (see Table 3). In line with H1a and H1b, the severe re‐traumatization group reported both the highest PTS severity and the greatest PTG compared to all other groups. Additionally, the mild re‐traumatization group (*M* = 0.95, *SE* = 1.07) also reported significantly higher PTG than the at‐risk‐only group (*M* = 0.58, *SE* = 0.94), *p* = .008, 95% CI [0.07, 0.67]. No significant differences were observed among the at‐risk only, at‐risk with past exposure, and mild re‐traumatization groups for PTS.

**TABLE 2:** Probable PTSD classification across the five groups.

PTSD	At‐risk only	At‐risk with past exposure	Recent exposure only	Mild re‐traumatization	Severe re‐traumatization
*n*	36	21	8	63	89
%	25.71	25.71	29.63	32.14	51.74
Missing	0	0	0	1	9

Abbreviation: PTSD, post‐traumatic stress disorder.

**TABLE 3:** Means, standard deviations, and one‐way analyses of variance across the groups at T1.

	At‐risk only	At‐risk with past exposure	Recent exposure only	Mild re‐traumatization	Severe re‐traumatization	*F*(3,592)	*η* ^2^
	*M* (*SD*)	*M* (*SD*)	*M* (*SD*)	*M* (*SD*)	*M* (*SD*)		
Identification	3.50 (2.02)	3.74 (1.92)	3.93 (2.22)	3.76 (1.78)	4.92 (1.91)	13.41***	.08
SSE	2.54 (1.70)	2.84 (1.80)	2.42 (1.80)	2.86 (1.52)	4.18 (1.85)	22.64***	.13
PTS	1.20 (0.95)	1.30 (0.93)	1.29 (1.03)	1.37 (0.86)	1.95 (1.04)	14.90***	.09
PTG	0.58 (0.94)	0.67 (0.84)	0.87 (1.12)	0.95 (1.07)	1.79 (1.27)	30.18***	.17

Abbreviations: PTG, post‐traumatic growth; PTS, post‐traumatic stress; SSE, sense of shared experience.

*** *p* < .001.

#### T1 and T2

A repeated measures MANOVA revealed a significant interaction between time and flood exposure group membership, Wilks' Λ = 0.92, *F*(16, 939) = 1.73, *p* = .037, *η* ^2^ = 0.02. In addition, there were significant main effects of the type of flood exposure group, Wilks' Λ = 0.85, *F*(16, 939) = 3.14, *p* < .001, *η* ^2^ = 0.04, and time, Wilks' Λ = 0.96, *F*(5, 306) = 2.94, *p* = .021, *η* ^2^ = .04, on the combined dependent variables. Follow‐up univariate tests showed that changes over time were not statistically significant for PTS, *F*(1, 310) = 2.79, *p* = .096, *η* ^2^ = .01, or for PTG, *F*(1, 310) = 0.43, *p* = .512, *η* ^2^ = 0.001, suggesting relative stability in symptom and growth levels across the two measurement points.

**FIGURE 1:**
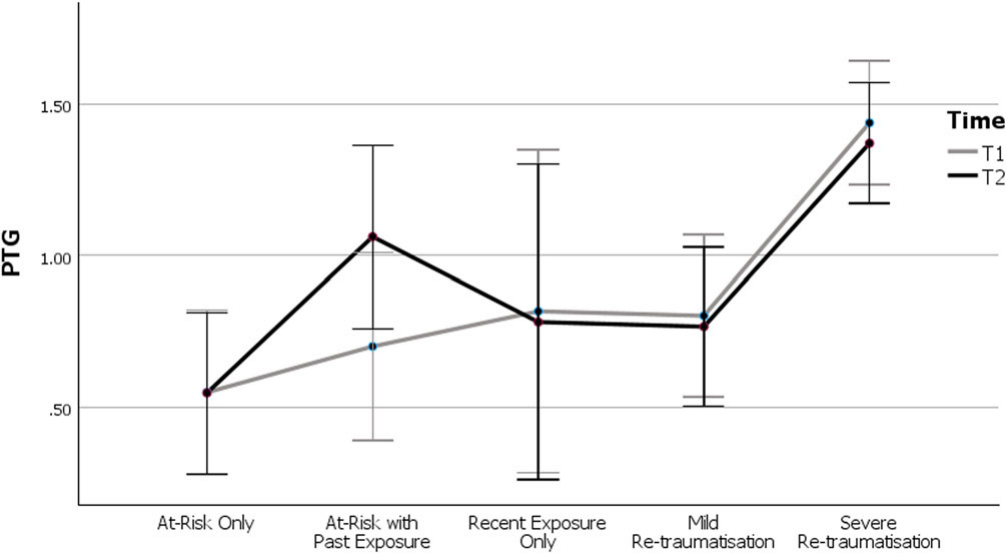
Estimated marginal means of post‐traumatic growth with people affected by flood across groups at two time points. *Note.* Error bars ±2 SE.

Between‐group comparisons, however, revealed robust differences. Differences between flood exposure groups were significant for PTG, *F*(4, 310) = 9.81, *p* < .001, *η* ^2^ = .11, but not for PTS, *F*(4, 310) = 1.93, *p* = .105, *η* ^2^ = .02. Post hoc comparisons showed that the severe re‐traumatization group (*M* = 3.60, *SE* = 0.14) reported significantly higher levels of PTG than in the mild re‐traumatization (*M* = 2.65, *SE* = 0.18) *p* < .001, 95% CI [0.21, 1.03], at‐risk with past exposure (*M* = 2.65, *SE* = 0.18) *p* = .012, 95% CI [0.08, 0.96], and the at‐risk only group (*M* = 2.63, *SE* = 0.18) *p* < .001, 95% CI [0.45, 1.25]. The mild re‐traumatization, at‐risk with past exposure, and at‐risk groups did not differ significantly from each other (see Figure 1). The results provided longitudinal support for Hypothesis 1b, as the severe re‐traumatization group consistently reported the highest levels of PTG across time points. In contrast, Hypothesis 1a was not supported, because PTS levels remained stable over time and did not differ significantly between the five exposure groups.

### Gained social identification and shared experience across flood exposure groups

#### T1

Levels of identification with flood‐affected people varied significantly across groups (see Table 3). Post hoc Games‐Howell comparisons showed that participants in the severe re‐traumatization group reported significantly higher identification with flood‐affected people (*M* = 4.92, *SE* = 1.91) than those in the at‐risk only group (*M* = 3.50, *SE* = 2.07), *p* < .001, 95% CI [0.81, 2.04], the at‐risk with past exposure group (*M* = 3.74, *SE* = 1.92), *p* < .001, 95% CI [0.48, 1.89], and the mild re‐traumatization group (*M* = 3.76, *SE* = 1.78), *p* < .001, 95% CI [0.63, 1.70]. SSE also varied depending on the flood exposure (see Table 3). The severe re‐traumatization group indicated a greater SSE than all other groups, *p*_*s*_ < .001.

#### T1 and T2

MANOVA's follow‐up univariate ANOVAs indicated that time had a significant effect on identification with flood‐affected people, (T1: *M* = 4.05, *SE* = 0.13; T2: *M* = 3.73, *SE* = 0.13), *F*(1, 310) = 7.00, *p* = .009, *η* ^2^ = 0.02, with identification decreasing across the two measurement points. No significant time effects were observed for SSE, *F*(1, 310) = 0.17, *p* = .683, *η* ^2^ = 0.001.

**FIGURE 2:**
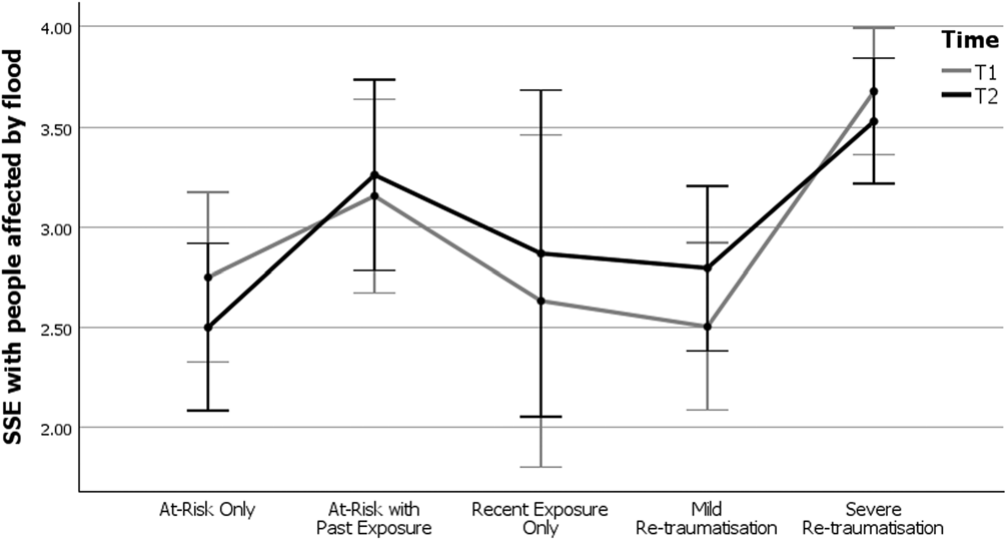
Estimated marginal means of sense of shared experience with people affected by flood across groups at two time points. *Note.* Error bars ±2 SE.

Between‐subjects tests confirmed significant group differences in SSE, *F*(4, 310) = 6.90, *p* < .001, *η* ^2^ = .08, but not in identification with flood‐affected people, *F*(4, 310) = 1.92, *p* = .107, *η* ^2^ = .02, or in PTS, *F*(4, 310) = 1.93, *p* = .105, *η* ^2^ = .02. Post hoc Games‐Howell analyses indicated that the severe re‐traumatization group maintained higher SSE compared to both the mild re‐traumatization group, *p* < .001, 95% CI [0.36, 1.55] and the at‐risk only group, *p* < .001, 95% CI [0.36, 1.60]. The mild re‐traumatization and at‐risk groups did not differ significantly from each other (see Figure 2).

### Psychosocial pathways to PTS and growth over time

#### Cross‐lagged associations in the overall flood‐affected sample

The cross‐lagged model estimated on the overall sample showed an excellent fit to the data (see Table S2). Strong autoregressive effects were observed for all key variables, indicating substantial temporal stability between the two waves. With regard to cross‐lagged relationships, SSE at T1 predicted identification with flood‐affected people; however, the reverse effect was not observed (H2). SSE at T1 predicted higher PTG at T2, partially confirming H4. However, contrary to H4, SSE at T1 was not associated with later PTS. Contrary to H3, identification at T1 did not predict changes in either PTS or PTG over time (see Table S2).

#### Cross‐lagged associations across flood exposure groups

As mentioned earlier, in addition to testing our hypotheses in the full sample, we also conducted between‐group analyses comparing participants with different levels of flood exposure. The multi‐group cross‐lagged panel model showed excellent fit to the data: *χ* ^2^(5) = 7.16, *p* = .209, CFI = 0.998, TLI = 0.935, RMSEA = 0.037, 90% CI [0.00, 0.09], SRMR = 0.005. Given the very small sample size (*n* = 17), results for the recent exposure‐only group were not interpreted. Full parameter estimates for the other groups are presented in Table 4.

**TABLE 4:** Direct relationships between variables in cross‐lagged regression: multi‐group analysis.

IV	DV	At‐risk only	At‐risk with past exposure	Resent exposure only	Mild re‐traumatization	Severe re‐traumatization
Identification (T1)	SSE (T2)	−0.15 (0.11)	0.12 (0.13)	−0.04 (0.37)	0.18 (0.10)	0.24** (0.09)
	PTS (T2)	−0.01 (0.06)	0.07 (0.07)	0.15 (0.18)	−0.02 (0.05)	−0.07 (0.05)
	PTG (T2)	0.04 (0.05)	0.03 (0.10)	−0.40*** (0.10)	0.08 (0.05)	0.04 (0.06)
	Identification (T2)	0.33** (0.13)	0.53*** (0.11)	0.30 (0.24)	0.37*** (0.10)	0.51*** (0.10)
SSE (T1)	Identification (T2)	0.19 (0.16)	0.31** (0.11)	0.20 (0.23)	0.09 (0.13)	−0.004 (0.11)
	PTS (T2)	−0.04 (0.08)	−0.04 (0.08)	−0.07 (0.18)	0.12* (0.06)	0.05 (0.06)
	PTG (T2)	0.03 (0.06)	0.23* (0.10)	0.31** (0.10)	0.18** (0.07)	0.07 (0.06)
	SSE (T2)	0.60*** (0.13)	0.31* (0.14)	0.46 (0.36)	0.39** (0.12)	0.24* (0.10)
PTS (T1)	Identification (T2)	0.05 (0.25)	−0.22 (0.19)	−0.11 (0.41)	−0.19 (0.25)	0.35* (0.17)
	SSE (T2)	−0.11 (0.20)	0.41 (0.24)	0.51 (0.63)	0.20 (0.24)	0.28 (0.15)
	PTG (T2)	0.003 (0.10)	0.14 (0.17)	0.53** (0.17)	−0.16 (0.12)	0.40*** (0.09)
	PTS (T2)	0.48*** (0.12)	0.44** (0.13)	0.12 (0.31)	0.52*** (0.11)	0.63*** (0.08)
PTG (T1)	Identification (T2)	−0.03 (0.25)	−0.23 (0.23)	0.97*** (0.27)	0.42* (0.20)	0.09 (0.14)
	SSE (T2)	0.06 (0.20)	0.39 (0.29)	0.43 (0.41)	−0.02 (0.19)	0.19 (0.12)
	PTS (T2)	0.18 (0.13)	−0.002 (0.16)	0.11 (0.20)	0.06 (0.09)	−0.01 (0.07)
	PTG (T2)	0.52*** (0.10)	0.35 (0.21)	0.88*** (0.11)	0.49*** (0.10)	0.33*** (0.07)

*Note*: Coefficients with standard deviations in brackets. SSE, sense of shared experience with people affected by the flood. PTG, post‐traumatic growth; PTS, post‐traumatic stress; SSE, sense of shared experience.

*** *p* < .001,

** *p* < .01, and

* *p* < .05.

Among the severe re‐traumatization group, identification with flood‐affected people at T1 predicted SSE at T2 (H2), *b* = 0.24, *SE* = 0.09, *p* = .006. In contrast, among those at risk with past exposure, SSE predicted later identification (H2), *b* = 0.31, *SE* = 0.11, *p* = .006. SSE with flood‐affected people predicted higher PTG in the group at risk with past exposure (H4), *b* = 0.23, *SE* = 0.10, *p* = .026, and the mild re‐traumatization group, *b* = 0.18, *SE* = 0.07, *p* = .007. Additionally, in the mild re‐traumatization group SSE also predicted PTS (H4), *b* = 0.12, *SE* = 0.06, *p* = .031. Identification was predicted by PTS in the severe re‐traumatization group, *b* = 0.35, *SE* = 0.17, *p* = .037, and by PTG in the mild‐traumatization group, *b* = 0.42, *SE* = 0.20, *p* = .034.

## DISCUSSION

The present study examined how individuals' history of flood exposure relates to their social identification and SSE with flood‐affected people, as well as to post‐traumatic outcomes. Using a quasi‐experimental design, we compared participants with different levels and histories of flood exposure and examined how these differences were associated with psychosocial responses over time. Cross‐lagged analyses further allowed us to investigate the temporal relationships among social identification, SSE, and post‐traumatic outcomes across two time points.

Overall, the results provided mixed support for our hypotheses. Hypotheses H1a and H1b received support at the first measurement, as PTS and PTG were most pronounced among participants reporting severe re‐traumatization. In the longitudinal analyses, the participants in the severe re‐traumatization group consistently reported the highest level of PTG (H1b), but hypothesis 1a was not supported by our data, as PTS levels did not differ between groups over time.

The result that participants in the severe re‐traumatization group reported the highest prevalence of probable PTSD and PTG at T1 reflects the paradox of trauma exposure, according to which repeated adversity may intensify distress and also foster growth (Calhoun & Tedeschi, 2006; Warsini et al., 2022). These results align with prior evidence that cumulative exposure can exacerbate PTS (French et al., 2019) yet also create opportunities for cognitive reappraisal and growth through shared struggle and strengthened social bonds (Craig et al., 2024). At the same time, our longitudinal analyses revealed that while PTG trajectories over time diverged by exposure group, PTS trajectories did not. This suggests that distress may stabilize, whereas growth remains more sensitive to exposure history.

Both social identification and SSE differed across flood exposure groups. However, the longitudinal analyses indicated no differences in social identification between groups over time, but the severe re‐traumatization group consistently showed the highest levels of SSE. Identification with flood‐affected people varied significantly across exposure groups, with those experiencing severe re‐traumatization reporting the highest levels of group identification. This finding suggests that repeated trauma may intensify social identity processes, potentially because shared hardship reinforces a sense of solidarity and belonging (Ntontis et al., 2020). Similarly, the SSE was consistently the highest among this group at both time points, indicating that perceiving commonality with others with similar experiences becomes especially pronounced in contexts of cumulative trauma. In other words, repeated flooding experience appears to reinforce not only individual identification with the affected community but also the awareness that one's experiences are collectively shared, which may serve as a key psychosocial resource. Interestingly, the effect of time was only significant with regard to identification with flood‐affected people. This suggests that regardless of flood exposure, people came to see themselves less as part of the group of flood‐affected individuals as time went on. In contrast, the SSE with flood‐affected people remained stable. This may reflect the difficulty of sustaining explicit identity labels as the immediate salience of disaster recedes, whereas SSE, anchored in lived experience, persists as a more enduring psychosocial resource. This distinction has practical implications: early interventions may benefit from fostering identification, while sustained support should leverage SSE as a long‐term resilience mechanism.

In Hypothesis 2, we expected a mutual reinforcement of social identification and SSE over time. The analyses provided an interesting insight: this relationship appears to depend on individuals' prior experiences. Specifically, identification with those affected by the flood predicted SSE only in the severe re‐traumatization group, whereas SSE predicted identification among participants at risk with previous traumatic experiences. Moreover, analyses conducted on the overall sample indicated a unidirectional effect, whereby SSE predicted identification (see Table S2). The longitudinal analyses provide important insights into how emerging social identification and SSE contribute to post‐traumatic outcomes following flood exposure. Among individuals experiencing severe re‐traumatization, group identification predicted SSE, suggesting that repeated trauma strengthens the identity‐to‐shared experience pathway. In contrast, for those at risk with past flood exposure, SSE predicted identification, indicating that commonality may be the gateway to the development of a group‐based identity.

We expected that the protective social processes, specifically higher levels of identification (H3) and SSE (H4) would be related to lower PTS and higher PTG. In the analyses conducted on the overall sample, we found support only for the role of SSE. Specifically, higher levels of SSE at T1 predicted higher PTG at T2 but were not associated with later PTS. Group identification did not predict changes in either PTS or PTG over time. These results highlight the role of perceived shared experience as a predictor of PTG over time. While traumatic events are often associated with distress and loss, they may simultaneously create opportunities for the emergence of new social identifications that support meaning‐making and PTG. In line with social identity approaches to collective adversity (e.g., Drury et al., 2019; Muldoon et al., 2025), identifying with others who share similar experiences may transform individual trauma into a collectively understood event, fostering solidarity and shared narratives of resilience. In this way, new group identification can serve as a pathway through which individuals derive positive psychological change following adversity.

More nuanced patterns emerged across exposure groups. Among those with past exposure to the disaster who were currently at risk, SSE predicted both stronger identification and greater PTG, suggesting that these mechanisms play a more adaptive role when the immediate threat is less acute. By contrast, in the mild re‐traumatization group, shared experience predicted both PTG and PTS, underscoring its potentially ambivalent role in shaping the trajectories of both recovery and distress. These findings extend the SIMTIC (Muldoon et al., 2019) by suggesting that the psychological processes linking collective trauma to identity formation may depend on individuals' trauma exposure histories. While severe or repeated trauma appears to strengthen group identification through shared distress, in contexts of milder or more distant exposure, SSE may play a stronger role in catalyzing identity formation and PTG. Importantly, SSE emerged as a stronger predictor of PTG across groups than group identification, underscoring the importance of shared experiences as a psychosocial resource in the aftermath of collective adversity.

The present study also extends the SIMTIC (Muldoon et al., 2019) to a new empirical context. While previous research has largely examined these processes in situations involving interpersonal or conflict‐related trauma, natural disasters represent a distinct form of collective adversity. Moreover, quantitative research examining the role of SSE in disaster contexts remains limited. By investigating SSE and social identification longitudinally among individuals affected by flooding, our study provides new empirical evidence on how shared experiences can contribute to psychosocial adaptation following natural disasters.

Although our study focused on flooding, these findings likely have broader implications for other natural disasters where individuals often confront traumatic events collectively. In such situations, perceiving that others have undergone similar experiences may facilitate meaning‐making and growth, even when it does not necessarily reduce distress.

As climate change amplifies the frequency and intensity of floods and other natural disasters, building psychosocial resilience becomes an essential component of climate change adaptation and public health planning. For public health and disaster response, our findings underline the importance of considering exposure histories when designing psychosocial interventions and disaster recovery programs. Strengthening social identification and recognition of shared experience may foster personal resilience and growth, but such interventions must also consider the risk that shared distress can exacerbate psychological strain in certain groups. Public health strategies should therefore approach disaster recovery not only through the lens of individual trauma but also as a socially embedded process shaped by collective identities and shared meanings. In practice, interventions could include facilitated community meetings where affected residents share experiences of the disaster, peer‐led recovery groups connecting individuals with similar exposure histories, or community storytelling initiatives that help transform individual experiences into shared narratives of resilience.

## LIMITATIONS AND FUTURE DIRECTIONS

While the present study offers important insights into psychosocial pathways to post‐traumatic outcomes in the aftermath of repeated flooding, several considerations should be kept in mind. The two‐wave longitudinal design provided a rare opportunity to track change over time, yet additional measurement points would allow for a more nuanced understanding of how PTS, growth, and social identity processes unfold across different stages of recovery. Second, the reliance on self‐report measures raises concerns about common‐method bias and social desirability, particularly in assessing sensitive constructs such as trauma symptoms and perceived growth. Future research could integrate clinical assessments, qualitative accounts, or physiological indicators to capture a broader range of experiences. Finally, while we managed to reach a rare and sensitive population, whose members are often reluctant to research because of the distressing nature of their experiences, the overall sample was moderate, and certain subgroups, such as those recently exposed to flooding, were particularly small, representing a potential limitation of the study. Replication in larger samples across diverse disaster contexts is necessary.

## CONCLUSION

The present research highlights the importance of social identity processes in shaping psychological responses to natural disasters. Although flood exposure was associated with both distress and growth, a SSE with other affected individuals appeared as a particularly stable and meaningful psychosocial resource that supported PTG over time. Differences between exposure groups further demonstrate that the role of social identity processes varies depending on individuals' trauma histories. The findings suggest that post‐disaster interventions may benefit from fostering the SSEs among affected communities. Importantly, such initiatives should also account for variation in prior trauma exposure to ensure that support is appropriately tailored to individuals' needs.

## CONFLICT OF INTEREST STATEMENT

The authors have no known conflict of interest to disclose.

## ETHICS STATEMENT

The studies were conducted in accordance with the Declaration of Helsinki, and the protocol was approved by the Scientific Research Ethics Committee of the Institute for Social Studies at the University of Warsaw (decision code: 10/24).

## Supporting information


**Table S1** *Comparison of Demographic Characteristics and Key Variables at T1 Between Those who Completed the Follow‐up and Those Who Did Not*.
**Table S2** *Model Fit and Direct Relationships between Key Variables in Cross‐lagged Regression in the Overall Sample*.
**Table S3** *Correlations between Key Variables at T1 and T2 in the Overall Sample*.

## Data Availability

The data that support the findings of this study are openly available in the Open Science Framework at https://osf.io/6p8vb/overview?view_only=43d5b48907e54cc39f548d40570326af.
