# Supplementary material for: Gained social identification and sense of shared experience after flood exposure: Psychosocial pathways to post‐traumatic symptoms and growth
Source: Appl Psychol Health Well Being. 2026 Jul 18;18(4):e70193. doi: 10.1111/aphw.70193 (PMC13379722; doi:10.1111/aphw.70193)
Supplement: Supplementary file 1 — Table S1 Comparison of Demographic Characteristics and Key Variables at T1 Between Those who Completed the Follow‐up and Those Who Did Not. Table S2 Model Fit and Direct Relationships between Key Variables in Cross‐lagged Regression in the Overall Sample. Table S3 Correlations between Key Variables at T1 and T2 in the Overall Sample. [file APHW-18-0-s001.docx]

**Supplementary materials**

**Table S1**

*Comparison of Demographic Characteristics and Key Variables at T1 Between Those who Completed the Follow-up and Those Who Did Not*

| Variables at T1 | Completed follow-up, *N*=315 | | Did not completed  follow-up, *N*=315 | | *t-test* | *p* |
| --- | --- | --- | --- | --- | --- | --- |
|  | *M* | *SD* | *M* | *SD* |  |  |
| Age | 46.52 | 15.06 | 40.16 | 13.21 | 5.63 | <.001 |
| Identification | 4.06 | 1.93 | 3.98 | 2.04 | 0.50 | .617 |
| SSE | 3.10 | 1.76 | 3.15 | 1.89 | -0.36 | .721 |
| PTS | 1.32 | 0.93 | 1.64 | 1.03 | -4.03 | <.001 |
| PTG | 0.97 | 1.15 | 1.15 | 1.20 | -1.88 | .061 |

**Table S2**

*Model Fit and Direct Relationships between Key Variables in Cross-lagged Regression in the Overall Sample*

| Model fit | χ²(1) = 0.25, *p* = .615, CFI = 1.000, TLI = 1.028,  RMSEA = .000, 90% CI [.00, .12], SRMR = .003 | |
| --- | --- | --- |
| IV | DV | *B (SE)* |
| Identification (T1) | SSE (T2) | .10 (.05) |
|  | PTS (T2) | -.02 (.03) |
|  | PTG (T2) | .03 (.03) |
|  | Identification (T2) | .43***(.05) |
| SSE (T1) | Identification (T2) | .14* (.06) |
|  | PTS (T2) | .03 (.03) |
|  | PTG (T2) | .12*** (.03) |
|  | SSE (T2) | .38***(.06) |
| PTS (T1) | Identification (T2) | .04 (.10) |
|  | SSE (T2) | .20* (.10) |
|  | PTG (T2) | .15** (.06) |
|  | PTS (T2) | .51***(.05) |
| PTG (T1) | Identification (T2) | .18* (.09) |
|  | SSE (T2) | .18* (.08) |
|  | PTS (T2) | .05 (.05) |
|  | PTG (T2) | .45***(.05) |

**Table S3**

*Correlations between Key Variables at T1 and T2 in the Overall Sample*

|  | Identification (T1) | SSE (T1) | PTS (T1) | PTG (T1) | Identification (T2) | SSE (T2) | PTS (T2) | PTG (T2) |
| --- | --- | --- | --- | --- | --- | --- | --- | --- |
|  | *B (SE)* | *B (SE)* | *B (SE)* | *B (SE)* | *B (SE)* | *B (SE)* | *B (SE)* | *B (SE)* |
| Flood exposure | .24 (0.17) | .44**(.16) | .22**(.08) | .52***(.11) | -.03 (.13) | .16 (.12) | -.09 (.07) | .08 (.07) |
| Identification (T1) | - | 1.91***(.22) | .51***(.11) | .96***(.14) | - | - | - | - |
| SSE (T1) | 1.91***(.22) | - | .68***(.10) | .92***(.13) | - | - | - | - |
| PTS (T1) | .51***(.11) | .68***(.10) | - | .43***(.07) | - | - | - | - |
| PTG (T1) | .96***(.14) | .92***(.13) | .43***(.07) | - | - | - | - | - |
| Identification (T2) | - | - | - | - | - | .75***(.12) | .16**(.06) | .32***(.07) |
| SSE (T2) | - | - | - | - | .75***(.12) | - | .13*(.06) | .25***(.07) |
| PTS (T2) | - | - | - | - | .16**(.06) | .13*(.06) | - | - |

*Notes.* ****p* <.001, ***p* <.01, **p* <.05.
